# Evaluation of Two Online Risk Prediction Models for the Mortality Rate of Acute Type A Aortic Dissection Surgery: The German Registry of Acute Aortic Dissection Type A Score and the European System for Cardiac Operative Risk Evaluation II

**DOI:** 10.3390/jcm12144728

**Published:** 2023-07-17

**Authors:** Ming Ma, Hailong Cao, Kai Li, Jun Pan, Qing Zhou, Xinlong Tang, Xichun Qin, Feng Zhu, Dongjin Wang

**Affiliations:** 1 Department of Thoracic and Cardiovascular Surgery, Nanjing Drum Tower Hospital, Affiliated Hospital of Medical School, Nanjing University, Nanjing 210008, China; 2 Department of Thoracic and Cardiovascular Surgery, Nanjing Drum Tower Hospital, Clinical College of Nanjing Medical University, Nanjing 210008, China; 3 Institute of Cardiothoracic Vascular Disease, Nanjing University, Nanjing 210008, China

**Keywords:** aortic dissection, EuroSCORE II, GERAADA score, online risk prediction models, surgery mortality

## Abstract

EuroSCORE II is one of the most widely utilized cardiovascular surgery risk scoring systems. Recently, a new online score calculator, namely the German Registry of Acute Aortic Dissection Type A (GERAADA), was launched to predict 30-day surgical mortality for acute type A aortic dissection (ATAAD) patients. The aim of this study is to evaluate the predictive performance of these two scores. We calculated the two scores for 1346 ATAAD patients from January 2012 to December 2021. The overall performance was evaluated using Brier scores and Hosmer-Lemeshow statistics. Receiver Operating Characteristic (ROC) curves were employed to assess diagnostic ability, and the standardized mortality ratio (SMR) was utilized to evaluate calibration. The GERAADA score and EuroSCORE II predicted 30-day mortality rates of 14.7% and 3.1%, respectively, while the observed rate was 12.5%. The predictive ability of EuroSCORE II (AUC 0.708, 95% CI: 0.664–0.792) was superior to that of the GERAADA score (0.648, 95% CI: 0.605–0.692). The GERAADA score had higher sensitivity but lower specificity than EuroSCORE II. And the GERAADA score may overestimate mortality (0.76, 95% CI: 0.65–0.89), while EuroSCORE II may underestimate the mortality rate (3.17, 95% CI: 2.92–3.44). The EuroSCORE II was superior in predicting surgical mortality among ATAAD patients. But the observed 30-day mortality rate certified a good calibration for the GERAADA score.

## 1. Introduction

Acute type A aortic dissection (ATAAD) is a critical surgical emergency that often presents with a range of life-threatening complications, including aortic valve insufficiency, pericardial tamponade, stroke, vascular rupture, and myocardial infarction [1]. Without prompt surgical intervention, the early mortality rate can escalate to as high as 1–2% per hour within 48 h of onset [2]. The primary objective of ATAAD surgery is to repair the aortic rupture and enhance blood flow via procedures such as ascending aorta replacement, aortic arch replacement, and other surgical interventions [3]. In cases where patients exhibit inadequate perfusion of vital organs, targeted measures must be taken to improve organ perfusion [4]. As such, ATAAD surgery is associated with prolonged operative times and significant trauma, with postoperative mortality rates remaining high [5]. Whether all patients with TAAAD need emergency surgery is still controversial in the clinic. Timely detection of patients at higher risk of death so as to take early measures may be of great significance to them. Some preoperative risk factors are associated with postoperative mortality in patients with ATAAD [6,7,8]. The European System for Cardiac Operative Risk Evaluation II (EuroSCORE II) is a widely utilized scoring system for cardiovascular surgery, not specifically ATAAD surgery [9]. It is imperative to develop an accurate scoring system that can predict the perioperative risk of these patients. Recently, an online risk scoring system to predict the 30-day mortality rate after ATAAD surgery, so-called the German Registry of Acute Aortic Dissection Type A (GERAADA) score, was developed based on the degree of malperfusion and the anatomical characteristics of the disease [10]. This scoring system has been validated by several centers and has shown promising results [11,12,13]. However, further research is needed to fully evaluate its efficacy. The risk factors incorporated into this scoring system exhibit numerous similarities with EuroSCORE II. Both risk scoring systems possess potential advantages in assessing postoperative mortality in TAAAD, yet consensus on the superior predictive tool remains elusive. Thus, this article aims to evaluate the efficacy of the GERAADA score and EuroSCORE II in predicting mortality after ATAAD surgery by gathering data on ATAAD cases in our cardiovascular center.

## 2. Materials and Methods

### 2.1. Ethics Statement

The studies involving human participants were reviewed and approved by the institutional review board of Nanjing Drum Tower Hospital (2020-185-01). Due to the retrospective and noninterventional study design, written informed consent for participation in this study was not required. All patient data was kept confidential.

### 2.2. Population and Settings

In this retrospective cohort study conducted at Nanjing Drum Tower Hospital Clinical College of Nanjing Medical University in China, data were extracted from our cardiovascular database to identify 1710 patients with a diagnosis of ATAAD who underwent surgery between January 2012 and December 2021. The exclusion criteria included DeBakey type II and III aortic dissection, onset time greater than 14 days or unknown, traumatic aortic dissection, tumors located anywhere within the body, active infections or autoimmune diseases, diagnosed Marfan syndrome, NYHA functional class III–IV, and incomplete information. Ultimately, we collected data from 1346 consecutive adult ATAAD patients for further analysis.

### 2.3. Data Collection

Clinical information on patients was obtained by strictly reviewing medical records according to the inclusion criteria. All necessary data for GERAADA scoring (https://www.dgthg.de/de/GERAADA_Score, accessed on 1 January 2023) and EuroSCORE II (https://www.euroscore.org/, accessed on 1 January 2023) risk factors were extracted from the cardiovascular database. We collected comprehensive demographic and operative data, encompassing age, gender, medical history, tobacco and alcohol use, as well as surgical techniques employed. The primary study endpoint was defined as all-cause mortality within 30 days after surgery.

### 2.4. Definitions

All subjects with ATAAD had chest or abdominal computed tomography (CT) images available. The ATAAD diagnosis was made using Computed Tomography Angiography (CTA) in accordance with the 2014 European Society of Cardiology Guidelines for the Diagnosis and Treatment of Aortic Diseases. The time between symptom onset and treatment is closely linked to the ATAAD prognosis. Based on our database, we included patients with ATAAD at hyperacute and acute stages, as specified in the exclusion criteria above.

The online GERAADA score and the EuroSCORE II calculator both provide precise definitions for patient characteristics. However, there are still some risk factors that have not been clearly described in either the calculators or the original manuscript. For preoperative organ malperfusion, the GERAADA score has four clear classifications: none, coronary, visceral, and peripheral malperfusion. Nevertheless, the golden diagnostic criteria for organ malperfusion are not listed in the online calculator. Some studies have emphasized that malperfusion should be defined as inadequate perfusion to certain organs due to dissection. However, the presence of tissue necrosis and vital organ failure, known as malperfusion syndrome (MPS), is not always evident in these patients. Therefore, our study utilized signs, symptoms, physical examination, and laboratory data to identify patients with malperfusion. To be more precise, in cases of coronary malperfusion, we conducted a thorough review of the patients’ medical records before they were transferred to our facility and/or assessed for ischemic electrocardiographic changes and/or dynamic fluctuations in troponin levels. These patients were classified as “CCS angina class 4” or “recent MI” according to the EuroSCORE II online calculator. Visceral malperfusion was identified by elevated lactate levels (>1.8 mmol/L), liver enzyme abnormalities, and persistent abdominal pain with tenderness upon palpation. For peripheral malperfusion, the presence of sudden limb pain or loss of motor function accompanied by a pulse deficit is indicative.

### 2.5. Arch Surgical Approach

We chose different methods for aortic arch repair according to the patients’ anatomic indications. Partial aortic arch replacement was defined as conservative arch repair (CAR), and total arch replacement and arch stent procedures were defined as total arch repair (TAR) [14].

### 2.6. Deep Hypothermia Cardiac Arrest Method

The right axillary artery and femoral artery were the first choices for arterial cannulation. Based on the expected duration of the circulatory arrest and the intended surgical procedure, we decided to use antegrade cerebral perfusion (ACP) or no cerebral perfusion. Intraoperative cerebral oxygen testing was routinely used to assess the effectiveness of intraoperative cerebral perfusion. When the reading was below 20% of the baseline, we considered the cerebral perfusion to have had an effect. For an expected arrest time of more than 30 min, the target value of cerebral temperature (Nasopharyngeal temperature) was 18–20 °C. For an arrest time of less than 30 min, the target value was 20–22 °C. We used an ACP perfusion rate of 3–8 mL/kg/min [14].

### 2.7. Statistical Analysis

Gaussian numerical variables are represented as mean ± standard deviation, while other variables are presented as median (25–75th percentile) and categorical variables as number (percentage). Normality was assessed using the Shapiro-Wilk test. All *p* values were two-tailed, with an alpha level set at 0.05. The Brier score is utilized to demonstrate overall modal performance, as previously described. The Brier score is a quadratic scoring rule that ranges from 0 (indicating perfect prediction) to 0.25 for a non-informative model. Receiver operating characteristic (ROC) curves were utilized to assess the diagnostic ability of all scores in predicting mortality, with calibration achieved through the area under the receiver operating characteristic curve (AUC) and Hosmer-Lemeshow goodness-of-fit test for binary outcomes. The discriminative ability is deemed excellent when the AUC exceeds 0.80, very good if it surpasses 0.75, and acceptable if it goes beyond 0.70. The statistical significance of the difference between the AUCs of both the GERAADA score and EuroSCORE II was assessed using Delong’s test. Calibration was tested using a calibration plot with bootstraps of 1000 resamples, which described the degree of fit between actual and the two models-predicted mortality.

We have utilized the standardized mortality ratio (SMR) to assess the calibration of two scoring systems. The SMR represents the ratio between observed deaths during a specific period in our study and expected deaths over that same time frame. Deviations from an SMR value of 1 indicate either underestimation or overestimation of mortality by the model. Moreover, if the 95% confidence interval (CI) of SMR encompasses a value of 1.0, it indicates good calibration of the model. The data analysis was performed using R software (version 4.1.3), with R packages “pROC (version 1.18.0)”, “ResourceSelection (version 0.3-5)”, “RMS (version 6.3)”, and “ggplot2 (version 3.3.6)” utilized for basic statistics and figures.

## 3. Results

In the final analysis, we included 1346 consecutive adult patients with ATAAD who underwent surgical repair at our cardiovascular center between January 2012 and December 2021. Of these patients, 326 were female (24.2%), and the median age was 53 years (IQR = 44–63). Table 1 displays the preoperative risk factors required by the GERAADA score.

The preoperative characteristics and prevalence of risk factors among ATAAD patients, as well as the surgery details required for EuroSCORE II calculation, are presented in Table 2. The GERAADA score predicted a median mortality rate of 14.7% (IQR = 12.1–17.9%), while the median EuroSCORE II was 3.1% (IQR = 2.2–4.7%).

The 30-day mortality rate following surgery in our study was 12.5% (168/1346). The Brier scores for the GERAADA score and EuroSCORE II were 0.106 and 0.101, respectively, indicating acceptable overall performance for both scores. Hosmer-Lemeshow goodness-of-fit statistics confirmed good calibration for EuroSCORE II (*p* = 0.96), but not for the GERAADA score (*p* < 0.001).

**Figure 1:**
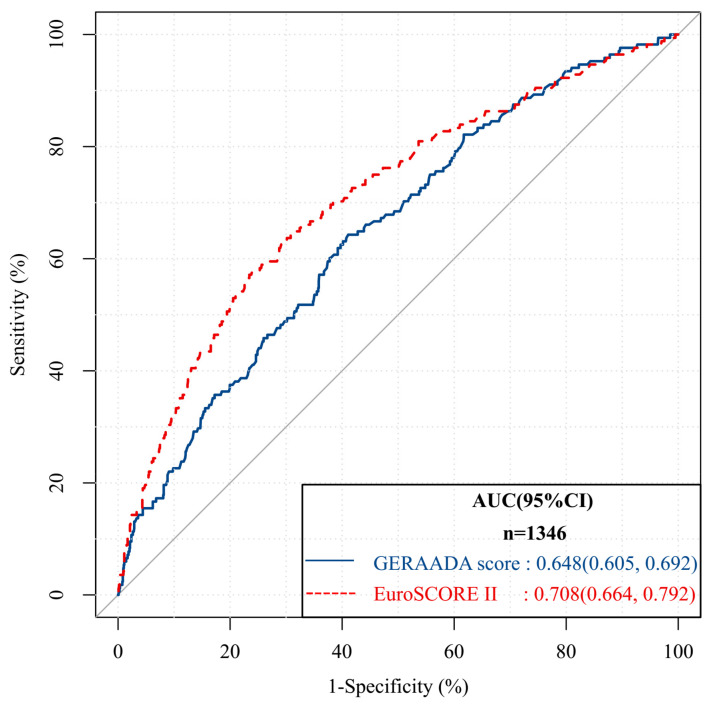
Comparison of the areas under the receiver operative characteristic curves of the German Registry of Acute Aortic Dissection Type A scores (AUC = 0.648, 95% CI: 0.605–0.692) and the European System for Cardiac Operative Risk Evaluation II (AUC = 0.708, 95% CI: 0.664–0.792). The comparison of the AUC (Delong test) showed a significant difference between two scores (z-statistic: −3.055, *p* = 0.002, 95% CI: −0.098, −0.021). AUC: areas under the curve; CI: confidence interval; EuroSCORE II: European System for Cardiac Operative Risk Evaluation II; GERAADA: German Registry of Acute Aortic Dissection Type A.

The EuroSCORE II calculation demonstrated a robust predictive ability for 30-day mortality (AUC 0.708, 95% CI: 0.664–0.792), whereas the GERAADA score exhibited an AUC of 0.648 (95% CI: 0.605–0.692, Figure 1). The comparison of the AUCs using the Delong test revealed a significant difference between the two scores (z-statistic: −3.055, *p* = 0.002, 95% CI: −0.098, −0.021).

In our cohort, the GERAADA score at the cutoff value of 14.5 and the EuroSCORE II calculation at 4.25 were the thresholds leading to the maximum summation of sensitivity and specificity in discriminating postoperative death in 30 days (refer to Table 3). Corresponding sensitivities for the GERAADA score and EuroSCORE II calculation were 66.7% and 51.2%, respectively, while specificities were 51.2% for the GERAADA score and 73.9% for the EuroSCORE II calculation, resulting in correct classification rates of 53.1% and 71.1%, respectively.

**Figure 2:**
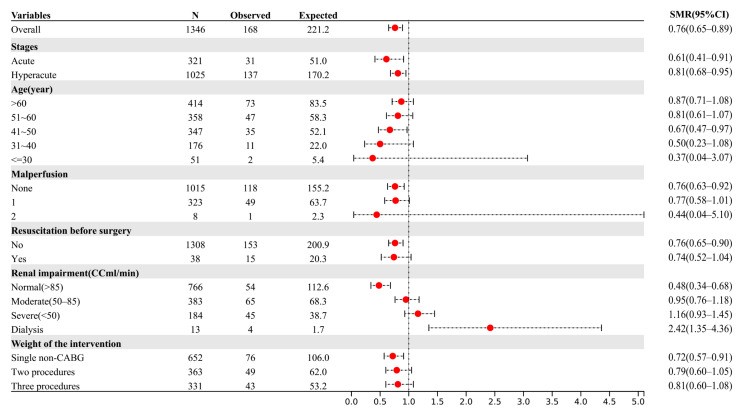
The forest plot showed overall mortality and the standardized mortality ratio of the GERAADA score according to subgroup. The solid vertical line indicates no difference between the observed and expected deaths of patients. Red dot represents the SMR of the variable. The black dotted line shows the 95% CI of the variable. AAS: acute aortic syndrome; AD: aortic dissection; CABG: coronary artery bypass grafting; CC: creatinine clearance; CI: confidence interval; GERAADA: German Registry of Acute Aortic Dissection Type A; IMH: intramural hematoma; SMR: standardized mortality ratio.

**Figure 3:**
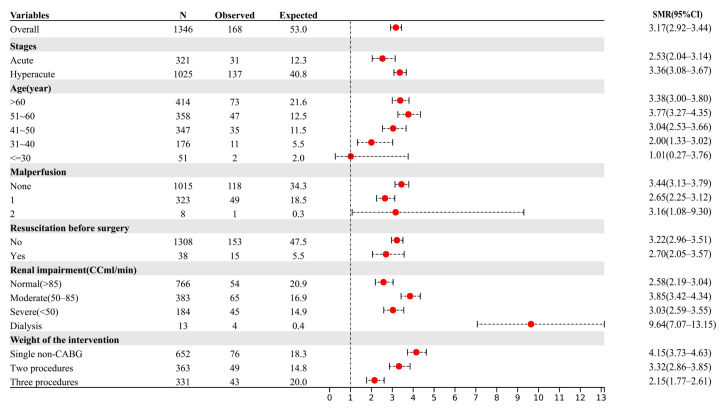
The forest plot showed the overall mortality and standardized mortality ratio of EuroSCORE II according to subgroup. The solid vertical line indicates no difference between the observed and expected deaths of patients. Red dot represents the SMR of the variable. The black dotted line shows the 95% CI of the variable. AAS: acute aortic syndrome; AD: aortic dissection; CABG: coronary artery bypass grafting; CC: creatinine clearance; CI: confidence interval; EuroSCORE II: European System for Cardiac Operative Risk Evaluation II; IMH: intramural hematoma; SMR: standardized mortality ratio.

**Figure 4:**
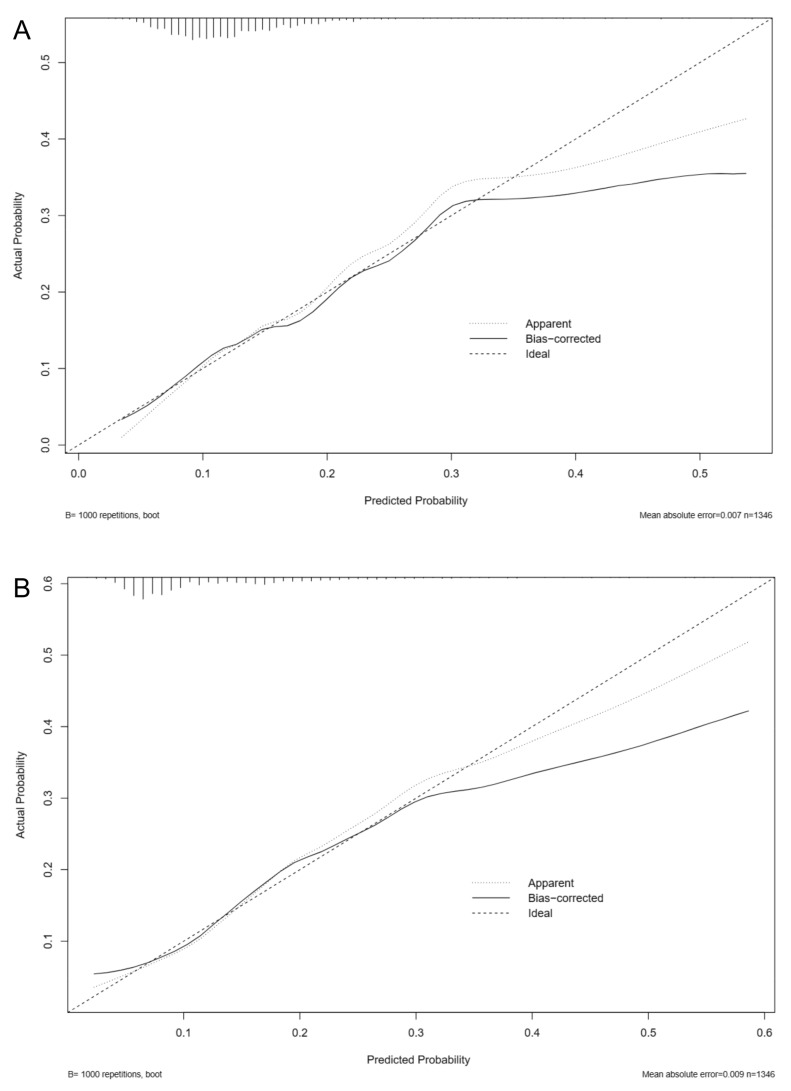
Calibration plot of the GERAADA score (**A**) and EuroSCORE II (**B**). The dotted line represents the performance of the two scores, whereas the solid line corrects for any bias in the two scores. The dashed line represents the reference line where each ideal score would lie.

The positive likelihood ratio for the GERAADA score was 1.37, while its negative likelihood ratio was 0.65. The SMR showed an overestimation of mortality for the GERAADA score (0.76, 95% CI: 0.65–0.89, Figure 2) and an underestimation of mortality for the EuroSCORE II calculation (3.17, 95% CI: 2.92–3.44, Figure 3). Figure 4 shows a calibration plot. This compares the prediction of mortality between the model prediction and the actual observation. The calibration plot revealed good predictive accuracy for the two models.

## 4. Discussion

In this study, we conducted a comparative analysis of the predictive accuracy of two scoring systems (the GERAADA score and EuroSCORE II) for mortality in patients undergoing ATAAD surgery using clinical data from over 1000 samples. Both scoring systems exhibited good overall performance, with the GERAADA score achieving a Brier score of 0.106 and EuroSCORE II achieving a Brier score of 0.101. The observed mortality rate was 12.5% (168/1346), while the GERAADA score predicted a mortality rate of 14.7% and EuroSCORE II predicted a low mortality rate of 3.1%. The SMR suggested that the GERAADA score may overestimate mortality (0.76, 95% CI: 0.65–0.89, Figure 2, Hosmer-Lemeshow goodness-of-fit statistics, *p* < 0.001), while EuroSCORE II may underestimate the mortality rate (3.17, 95% CI: 2.92–3.44, Figure 3, Hosmer-Lemeshow, *p* = 0.96). Additionally, EuroSCORE II exhibited superior discriminative power (AUC 0.708, 95% CI: 0.664–0.792) compared to the GERAADA score (AUC 0.648, 95% CI: 0.605–0.692), with a z-statistic of −3.055 and a *p*-value of 0.002. It should be noted that while the GERAADA score demonstrated higher sensitivity but lower specificity in identifying postoperative mortality within 30 days, as shown in Table 3. Based on the findings of our study, both scoring systems demonstrated advantages and disadvantages; therefore, it remains challenging to determine which system outperforms the other.

Although the GERAADA scoring system is commendable as the first online calculator for predicting 30-day mortality in patients undergoing ATAAD surgery, there are still many aspects of the scoring system that require further improvement. As previously mentioned, the online calculator does not offer the gold standard diagnostic criteria for evaluating organ malperfusion. This may potentially cause confusion among users of the GERAADA scoring system as they could establish their own diagnostic criteria based on previous research and self-experiences, leading to inconsistent definitions of organ malperfusion and ultimately impacting the final score of the calculator. The diagnosis of malperfusion is primarily based on clinical history, physical examination, laboratory tests, and imaging. CTA is commonly utilized for diagnosing aortic dissection and aiding in the identification of perfusion syndrome [15]. Cho et al. [16] found that the absence of malperfusion did not exhibit a statistically significant difference in early and long-term mortality compared to subclinical malperfusion, whereas clinical malperfusion significantly increased the risk of adverse outcomes. Yang et al.’s study [17] showed that the presence of malperfusion syndrome, resulting from ATAAD combined with end-organ dysfunction, is a significant adverse prognostic factor for long-term survival, particularly in cases involving mesenteric malperfusion. The strategy of immediate reperfusion, stabilization, and planned open aortic reconstruction still carries a high risk of early mortality (38%). Patients who survived the initial malperfusion and underwent delayed open aortic reconstruction exhibited no adverse effects, with similar surgical and late survival rates comparable to those without complex dissection. Malperfusion appears to be a fundamental prognostic risk factor for ATAAD after surgical repair, and the GERAADA score is currently the sole short-term mortality quantitative scoring system that incorporates malperfusion as a variable. It is imperative to define malperfusion, particularly in terms of malperfusion grading, to enhance the calculative clarity of the GERAADA score.

Furthermore, the GERAADA score solely focuses on preoperative risk factors and does not incorporate intraoperative variables that may affect the ATAAD patients’ surgical prognosis. A more extensive replacement that involves the aortic root, arch, or both could add operative complexity, surgery time, and potentially circulatory arrest time. Involvement of the aortic arch by the AD can influence both interventional strategies and clinical outcomes. Total arch replacement (TAR) is safe in some single-center studies [18], but with higher rates of blood transfusion [19] and higher operative death than hemiarch in other databases (27% versus 16%, *p* < 0.001) [20]. Due to these conflicting research results, it needs more samples to assess the real predictive capacity of the score with the different surgery strategies.

Risk factors for ATAAD patients are multiple. For example, in-hospital mortality was more than four times higher in patients who had aortic crossclamping time for more than 2 h. In addition, survivors who underwent surgical treatment had significantly shorter surgery times, statistically shorter cardiopulmonary bypass times, and received significantly fewer transfused packed red blood cells than nonsurvivors. The percentage of nonsurvivors who need additional coronary artery bypass surgery is also elevated [21]. Furthermore, preoperative cardiac function in ATAAD patients is a crucial determinant of in-hospital mortality [22,23] and is also a key factor affecting scores in EuroSCORE II, but its precise value remains undefined in the GERAADA score.

### Limitations

Although our clinical study includes over 1000 samples, we must acknowledge that there are still certain limitations. Firstly, in comparison to the GERAADA registry, our sample size was relatively small. Additionally, this study was conducted at a single center and requires further validation through prospective multicenter studies. Furthermore, the results of this study may be influenced by ethnic differences. And repeated enhanced CT examinations may further burden the patients, and clinical evaluation is convenient and effective, so the judgment of limb ischemia in our study is subjective to a certain extent, which is another limitation of our study. Due to the lengthy duration of this study, advancements in surgical techniques may introduce some deviations from the research findings.

## 5. Conclusions

Our research findings suggest that the GERAADA score, a newly developed online scoring system specifically designed for ATAAD, is not inferior to the EuroSCORE II score in predicting surgical mortality among patients undergoing ATAAD surgery. Although the GERAADA score still has some limitations that require further refinement and additional prospective clinical trials are necessary to fully assess its efficacy, as a novel tool for guiding preoperative clinical decision-making, it is certainly worth promoting.

## Figures and Tables

**Table 1:** Patient preoperative characteristics (the GERAADA) of the study population.

Characteristics	Number of Patients (%)
Age (year), media (IQR)	53 (44, 63)
Sex (female)	326 (24.2)
Resuscitation before surgery	38 (2.8)
Previous cardiac surgery	14 (1.0)
Intubation/ventilation at referral	38 (2.8)
Catecholamines at referral	38 (2.8)
Aortic valve regurgitation	
◆ none	978 (72.7)
◆ grade I or II	162 (12.0)
◆ grade III order IV	206 (15.3)
Preoperative organ malperfusion	
◆ none	1015 (75.4)
◆ coronary malperfusion	13 (1.0)
◆ visceral malperfusion	17 (1.3)
◆ peripheral malperfusion	309 (23.0)
Preoperative hemiparesis	7 (0.5)
Extension of disscection	
◆ aortic arch	1346 (100.0)
◆ supraaortic vessels	205 (15.2)
◆ descending or further downstream	1346 (100.0)
Location of primary entry tear within aortic arch	418 (31.1)

GERAADA: German Registry of Acute Aortic Dissection Type A; IQR: interquartile range.

**Table 2:** Patient preoperative characteristics and surgery details (EuroSCORE II) of the study population.

Characteristics	Number of Patients (%)	Characteristics	Number of Patients (%)
Age (year), media (IQR)	53 (44, 63)	Weight of the operation	
Sex (female)	326 (24.2)	◆ Isolated CABG	0 (0)
Chronic pulmonary disease	54 (4.0)	◆ Single non CABG	653 (48.5)
Extracardiac arteriopathy	309 (23.0)	◆ 2 procedures	362 (26.9)
Poor mobility	7 (0.5)	Graft interposition + CABG	36 (2.7)
Previous cardiac surgery	14 (1.0)	Graft interposition + AVr/AVR	48 (3.6)
Active endocarditis	0 (0)	Graft interposition + MVr/MVR	2 (0.1)
Critical preoperative state	38 (2.8)	Graft interposition + TVr	5 (0.4)
Renal impairment (ml/min)		Total arch replacement	271 (20.1)
◆ Normal (>85)	766 (56.9)	◆ 3 procedures and more	331 (24.6)
◆ Moderate (50–85)	383 (28.5)	Graft interposition + AVR + CABG/AVr + MVr/CABG + TVr/MVR + TVr	7 (0.5)
◆ Severe (<50)	184 (13.7)	Bentall	160 (11.9)
◆ dialysis	13 (1.0)	Bentall + CABG/MVr/MVR/TVr	32 (2.4)
Diabetes on insulin	61 (4.5)	David	12 (0.9)
CCS angina class 4	13 (1.0)	David + CABG/MVr/MVR/TVr	2 (0.1)
Recent MI	13 (1.0)	Total arch replacement + Bentall	64 (4.8)
Surgery on thoracic aorta	1346 (100)	Total arch replacement + Bentall + CABG/MVr + TVr/TVr	6 (0.4)
Urgency		Total arch replacement + David	2 (0.1)
◆ urgent	517 (38.4)	Total arch replacement + CABG	18 (1.3)
◆ emergency	829 (61.6)	Total arch + AVr/MVr/MVR/TVr	21 (1.6)

AVr: aortic valve repair; AVR: aortic valve replacement; CABG: coronary artery bypass grafting; CCS: Canadian Cardiovascular Society; EuroSCORE II: European System for Cardiac Operative Risk Evaluation II; IQR: interquartile range; MI: myocardial infarction; MVr: mitral valve repair; MVR: mitral valve replacement; TVr: tricuspid valve repair.

**Table 3:** Diagnostic performance for predicting mortality using the GERAADA score compared to the EuroSCORE II calculation.

	Threshold	Specificity, %	Sensitivity, %	Accuracy, %	PLR	NLR	PPV, %	NPV, %
Patients (n = 1346)								
GERAADA score	14.5	51.2	66.7	53.1	1.37	0.65	16.3	91.5
EuroSCORE II	4.25	73.9	51.2	71.1	1.96	0.66	21.9	91.4

EuroSCORE II: European System for Cardiac Operative Risk Evaluation II; GERAADA: German Registry of Acute Aortic Dissection Type A; NLR: negative likelihood ratio; NPV: negative predictive value; PLR: positive likelihood ratio; PPV: positive predictive value.

## Data Availability

The datasets used and/or analyzed during the current study are available from the corresponding author on reasonable request.
